# Radiation Therapy for Palliation of Sarcoma Metastases: A Unique and Uniform Hypofractionation Experience

**DOI:** 10.1155/2010/927972

**Published:** 2010-03-08

**Authors:** Viacheslav Soyfer, Benjamin W. Corn, Yehuda Kollender, Haim Tempelhoff, Isaac Meller, Ofer Merimsky

**Affiliations:** ^1^Division of Oncology, Institute of Radiation Therapy, Tel-Aviv Sourasky Medical Center, 6 Weizmann Street, Tel-Aviv 64239, Israel; ^2^The Sackler School of Medicine, Tel-Aviv University, Tel-Aviv 69978, Israel; ^3^National Unit of Orthopedic Oncology, Tel-Aviv Sourasky Medical Center, 6 Weizmann Street, Tel-Aviv 64239, Israel; ^4^Unit of Bone and Soft Tissue Oncology, Division of Oncology, Tel-Aviv Sourasky Medical Center, 6 Weizmann Street, Tel-Aviv 64239, Israel

## Abstract

Radiotherapy (RT) is our preferred modality for local palliation of metastatic soft tissue sarcoma (STS). A short and intense course of RT is usually needed for rapid palliation and local control of metastatic disease. Seventeen patients at a median age of 61 had symptomatic metastatic sarcoma and required rapid palliation. The symptoms related to the metastases were either pain or discomfort. All patients were treated by a short and intensive course of administration: 39 Gy were given in 13 fractions of 3 Gy/day, 5 times a week. Median follow-up period was 25 weeks. The treatment was well tolerated. Acute side effects included grade one skin toxicity. No wound complications were noted among those undergoing surgery. Late side effects included skin pigmentation and induration of irradiated soft tissues. Durable pain control was achieved in 12 out 15 cases treated for gross metastases. Tumor progression was seen in the 3 other cases within a period of two to nine months. Among 5 lesions which were irradiated as an adjunctive treatment following resection, no local recurrence was observed. The results of this series, although limited in size, point to the safety and feasibility of hypofractionated RT for palliation of musculoskeletal metastases from sarcoma.

## 1. Introduction

Soft tissue sarcomas (STS) of the extremities in young or good-performance-status patients are currently approached by limb sparing surgery (LSS) followed by radiation therapy (RT) [1] . We have previously reported a series of 133 adult patients with intermediate or high-grade limb STS who were approached by LSS + RT [2]. Along with others, we have previously demonstrated that a schedule of 1.8 Gy/fraction, 5 fractions per week to a midplane dose of 63 Gy, or 70 Gy in the setting of marginal excision or involved margins, was feasible, tolerable and efficient [2]. Locally advanced cases, involving the neurovascular bundle or the underlying bone, may be treated with surgery following induction chemotherapy [3] or isolated limb perfusion with tumor-necrosis-factor-*α* [4], or by definitive RT [5] or by amputation [6]. Locally recurrent or metastatic disease may be palliated by systemic chemotherapy, surgery, and RT. Amputation has been advocated as a palliative procedure for symptomatic locally advanced disease that has already failed to respond to radiation therapy, chemotherapy, and limited surgery [7]. RT is our preferred modality for local palliation of metastatic disease, irrespective of whether systemic chemotherapy is employed. While a protracted course of RT may be given as postoperative adjuvant treatment, a short and intense course of RT is usually needed for rapid palliation and local control of metastatic disease. Our impression was that the commonly applied RT doses for palliation of other malignancies were not adequate for palliation of sarcoma metastases. Many patients required reirradiation of lesions (unpublished data). In this paper we report a feasibility study of 39 Gy administered in 13 fractions of 3 Gy each, over 13 working days (five days per week), in a group of patients with metastatic sarcoma.

## 2. Materials and Methods

### 2.1. Patients

Seventeen patients, 8 women and 9 men, at a median age of 61 years (range 53–95 years) had symptomatic metastatic sarcoma, and required rapid palliation. The types of sarcoma included liposarcoma (5 patients), pleomorphic sarcoma (3), chondrosarcoma (2), synovial sarcoma (1), bone sarcoma (1 Ewing sarcoma of pelvis, 1 leiomyosarcoma of bone), leiomyosarcoma (2), and unclassified sarcoma (2). In total there were 20 sites of involvement by metastatic disease: trunk (chest wall, groin, axilla)—13 cases, limb- 7 cases. The symptoms related to the metastases were either pain or discomfort in all the patients. In 15 cases the RT was the only modality for local palliation and in 5 cases RT was given following metastasectomy with close or involved margins.

### 2.2. Radiation Therapy

All patients underwent CT simulation (Phillips Brilliance Big Bore). The positioning and immobilization of patients was individualized as a function of tumor location. Beams were designed to achieve coverage of the clinical treatment volume (CTV). CTV included the gross tumor volume (GTV), that in cases of palliation mostly included the viable and visible tumor with margins of 1.5–2 cm, and in cases of postmetastasectomy treatment after wide or marginal resections, included the tumor in it's location before the surgery. In those cases the CTV was based on the preoperative CT scan or MRI and clips which surgeons placed at the tumor bed and its edges. Special attention in postoperative cases was devoted to the linear borders of the fields so as to be at least 4 to 5 cm beyond the edges of tumor bed. Treatment planning was carried out with the XiO system (CMS Corporation: St. Louis, MO, USA) and was according to requirements of ICRU-50. Before the start of treatment each patient had verification of portals (Elekta I-View GT- IVIEW02). The radiation was provided by ELEKTA linacs (Stockholm, Sweden) with energies of 6 or 18 MV. Patients were assessed for side effects and compliance with treatment on a weekly basis during therapy, at the end of treatment, and every 3 months thereafter. All patients were treated by a short and intensive course of administration; 39 Gy were given in 13 fractions of 3 Gy/day, 5 times a week. This fractionation regimen was selected to achieve a meaningful dose based on *α*-*β* modeling (*vide infra*).

## 3. Results

The median follow-up period was 25 weeks (range: 12–89 weeks). Thirteen fractions were given over 13–16 days. The treatment was well tolerated. Acute side effects included grade 1 skin reaction (faint erythema as per CTC Version 2.0) (16) in all cases. No wound complications were noted. Durable pain control was achieved in 12 out 15 cases treated for gross metastases. Tumor progression was seen in the 3 other cases within a period of two to nine months. Among the 5 lesions which were irradiated post-metastasectomy, no local recurrence was observed.

All patients were invited back for follow-up visits to the multidisciplinary sarcoma clinic on a quarterly basis where physical exam and review of imaging was performed (including RECIST criteria) (15). By evaluating tumor response in this manner we were able to determine time to local progression. 

**Figure 1:**
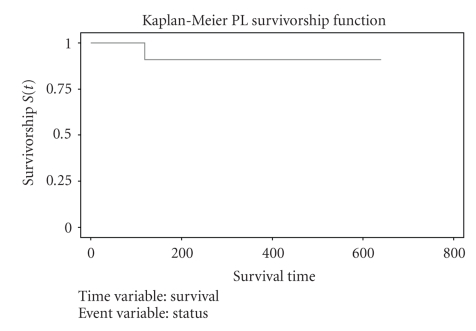


Fourteen patients were alive at the end of the observation period (Kaplan-Meir analysis—Figure 1).

## 4. Discussion

Sarcomas are usually considered, at best, moderately radio responsive tumors. RT doses at range of 60–70 Gy are usually needed to be delivered in order to eradicate microscopic disease, while doses of 50 Gy can yield similar results for other malignancies such as breast or rectal cancer. One of the biological characteristics of sarcoma cells is their relatively low (−0.5–5.4) *α*-*β* ratio [8] . This ratio, theoretically, may justify the use of larger-than-standard fractionation in order to achieve significant cell-kill by RT. We used a popular formula for standardization of different fractionation regimens: BED = ND × (1 + *D*/*α*-*β* ratio), when BED is dose equivalent to standard fractionation of 1.8–2 Gy, *N* is the number of fractions, and *D* is the given fraction dose [8]. To achieve the dose equivalent to 60–70 Gy we decided to use 13 fractions of mildly elevated daily dose of 3 Gy. Assuming the alpha- beta ratio of Sarcoma cells as 4 our calculation of BED is as following: 13 × 3 (1+ 3/4) = 68 Gy.

The results of this series, although limited in size, point to the safety and feasibility of hypofractionated RT for palliation of musculoskeletal metastases from sarcoma. The chosen fractionation regime appears to achieve adequate local and symptom control in the majority of patients during the short to medium term. While a protracted course of RT is applied for post-operative adjuvant RT as part of LSS approach, a short and intense course may be needed for local control and palliation. In the presence of metastatic disease, especially when the expected survival is limited, it is important to deliver the RT over a short period, and avoid the inconvenience associated with a multifractionated regimen of radiotherapy. This issue may be particularly true for elderly and debilitated patients, in whom a short course of RT may be the only practical option for a local palliation. All the patients received the RT during an acceptable period of time, and well tolerated the therapy. 

Hypofractionation as a part of neoadjuvant chemo-radiotherapy for STS was popularized by Eilber et al. [3], who applied a regimen of 10 fractions of 3.5 Gy each. The rationale was based on the observation that irradiated melanoma cell lines had a large initial shoulder on their survival curve. The potential radio resistance of melanoma and, by extension, sarcoma, was attributed to tumor cell capacity for sublethal damage repair, as implied by this shoulder [9]. 

The histological response of dose intense chemotherapy with preoperative hypofractionated radiotherapy in patients with STS was further investigated by Ryan et al. [10]. They reported a high rate of pathological tumor necrosis (95%) following an aggressive chemoradiotherapy regimen of 28 Gy administration in 8 fractions [10]. Other authors also justified an aggressive approach to palliate distant metastases; a wide local resection of the disseminated disease foci followed by adjuvant radiation therapy [11, 12]. Kepka et al. [5] described the results of radiation therapy as a sole treatment of gross disease in 112 patients [5]. Patients who received more than 63 Gy in standard fractionation of 1.8–2 Gy achieved best local control, disease free interval, and overall survival rate.

An animal model of a hypofractionation regimen for macroscopic soft tissue sarcomas was created and reported by Lawrence et al. [13]. Four fractions of 8 Gy were prescribed to 16 dogs with overt masses of STS. The overall response rate was 50% (one complete response) with minimal side effects. The median time to progression was 155 days and the median survival time was 309 days [13]. 

A potential use of hypofractionated RT is in elderly or debilitated population with STS. Patients with sarcoma of the extremity who have a good performance status may easily tolerate a protracted course of adjuvant RT following LSS, even if induction chemotherapy or tumor-necrosis factor via isolated limb perfusion had been primarily administered [2]. However, patients with low-performance status or with multiple comorbidities and mobility difficulties may be deemed medically unfit for the regimen proposed herein. While advanced age in the presence of a good performance status is not a limiting factor in the treatment strategy for STS (LSS and RT) [2], or in other diseases (e.g., non-Hodgkin's lymphoma [2] and lung cancer [14]), a patient with sarcoma and poor performance status or severe co-morbidities, might not be able to attend a long course of RT (5 times a week for a total of 6–8 weeks), and might even initially give up the important postoperative treatment. Furthermore, a prolonged hospitalization for RT may result in unnecessary hospital-acquired infections and social problems due to a change in the patient's close surroundings. Omitting RT in an LSS-approached patient increases the risk of local recurrence [1]. As an alternative to LSS without RT, amputation surgery may be suggested, hampering the quality of life even more, and rendering the patient bed-ridden for the rest of his life. A short and intense course of RT may be a logical and adequate solution to this dilemma.

The results reported offer encouraging data that can be applied to patients suffering from this daunting disease. Prospective studies could assess the role of hypofractionation and other palliative strategies among debilitated or elderly patients with STS.

## Figures and Tables

**Table 1:** Patient Characteristics.

Pt	Age	Sex	Site	Pathology	Indication of Treatment	DDD	TTLP	TTDP
1	53	F	Chest	LMC	Adjuvant to surgery	13		61
2	78	F	Thigh	Pleomorphic	Adjuvant to surgery	13		
3	65	M	Groin	HGSTS	Palliation	13		
4	71	M	Pelvis	Myxoid Chondrosarcoma	Palliation	13	266	
5	86	F	Calf	Chondrosarcoma	Palliation	13		
6	68	F	Pelvis	MFH	Palliation	13		413
7	41	M	Chest and thigh	SS	Palliation	13	196	247
8	59	F	Thigh	Bone LMS	Palliation	16		56
9	44	M	Chest	HGSTS	Palliation	13		180
10	49	M	Thigh	MFH	Adjuvant to surgery	15		48
11	51	F	Foot	HGSTS	Palliation	14		
12	50	M	Pelvis	LMS	Palliation	13		
13	60	M	Groin	LMS	Adjuvant to surgery	13		90
14	27	M	Pelvis	Ewing	Palliation	13		5
15	51	F	Chest	LMS	Palliation	13	12	12
16	80	F	Calf, groin, axilla	HGSTS	Palliation	13		
17	95	M	Thigh	LMS	Adjuvant to surgery	13		270

Key:Pt: Patient; F:Female; M: Male;LMS: Leiomyosarcoma; HGSTS: High Grade Soft Tissue Sarcoma; MFH: Malignant Fibro Hystiocytoma; SS: Synovial Sarcoma; DDD: Duration of dose delivery; TTLP: time to local progression; TTDM: time to distant progression (*the latter 3 parameters all measured in days*).
